# Late-Season Nitrogen Applications Increase Soybean Yield and Seed Protein Concentration

**DOI:** 10.3389/fpls.2021.715940

**Published:** 2021-10-06

**Authors:** Anuj Chiluwal, Erin R. Haramoto, David Hildebrand, Seth Naeve, Hanna Poffenbarger, Larry C. Purcell, Montserrat Salmeron

**Affiliations:** ^1^Department of Plant and Soil Sciences, University of Kentucky, Lexington, KY, United States; ^2^Department of Agronomy and Plant Genetics, University of Minnesota, Saint Paul, MN, United States; ^3^Department of Crop, Soil, and Environmental Sciences, University of Arkansas, Fayetteville, AR, United States

**Keywords:** soybean meal, bradyrhizobia soil inoculation, cover crop, cereal rye, oat, winter wheat

## Abstract

Low seed and meal protein concentration in modern high-yielding soybean [*Glycine max* L. (Merr.)] cultivars is a major concern but there is limited information on effective cultural practices to address this issue. In the objective of dealing with this problem, this study conducted field experiments in 2019 and 2020 to evaluate the response of seed and meal protein concentrations to the interactive effects of late-season inputs [control, a liquid *Bradyrhizobium japonicum* inoculation at R3, and 202 kg ha^−1^ nitrogen (N) fertilizer applied after R5], previous cover crop (fallow or cereal cover crop with residue removed), and short- and full-season maturity group cultivars at three U.S. locations (Fayetteville, Arkansas; Lexington, Kentucky; and St. Paul, Minnesota). The results showed that cover crops had a negative effect on yield in two out of six site-years and decreased seed protein concentration by 8.2 mg g^−1^ on average in Minnesota. Inoculant applications at R3 did not affect seed protein concentration or yield. The applications of N fertilizer after R5 increased seed protein concentration by 6 to 15 mg g^−1^, and increased yield in Arkansas by 13% and in Minnesota by 11% relative to the unfertilized control. This study showed that late-season N applications can be an effective cultural practice to increase soybean meal protein concentration in modern high-yielding cultivars above the minimum threshold required by the industry. New research is necessary to investigate sustainable management practices that increase N availability to soybeans late in the season.

## Introduction

Soybean is the most important source in the world when it comes to high-quality protein for livestock and poultry feed. While average soybean yields in the United States have increased at a rate of 28 kg ha^−1^ yr-^1^ between 1986 and 2019, soybean seed protein concentration has declined at a rate of 0.51 mg protein g^−1^ yr^−1^ during the same period (Naeve and Miller-Garvin, 2019). This trend is particularly concerning in northern U.S. states, which typically produce soybean with lower protein concentrations than southern U.S. states (Hurburgh Jr et al., 1990; Rotundo et al., 2016). However, despite the latitudinal trend in the U.S., regional studies indicated that greater spatial variability in protein concentration exists within a region than among regions and years (Rotundo et al., 2016). These results indicated that other factors such as site-specific nutrient and management factors could play an important role in driving the variability in seed protein concentration.

Late-season nitrogen (N) applications may be one cultural approach to increase seed protein concentration. The importance of N availability to soybean during reproductive growth is indicated by its high rates of N accumulation in the seed during the seed filling phase. For example, the crop requires 183 kg N ha^−1^ transported into its seeds to produce a grain yield of 3,339 kg ha^−1^ with 342 mg g^−1^ protein, based on the U.S. mean yield and seed protein concentration during 2015 to 2019 from Naeve and Miller-Garvin (2019). Therefore, high rates of N accumulation in the seed must come from mining of inorganic N mineralized from soil organic matter, biological N fixation, or from remobilization of N from vegetative tissues to the seed. While the availability of N during the seed filling phase may be critical, most studies have evaluated the impact of early-season N fertilizer applications on seed protein concentration rather than late-season applications (Osborne and Riedell, 2006; Ray et al., 2006; Wilson et al., 2014; Kaur et al., 2017). A meta-analysis conducted in 2009 found an average increase in protein concentration of +0.7% with N fertilization before flowering, and of +1.1% with applications during flowering and pod setting relative to the unfertilized control (Rotundo and Westgate, 2009). A recent study with high rates of N fertility (540–870 kg N ha^−1^) split over the growing season suggested that it may be possible to increase both soybean yield and seed protein concentration through increasing crop N availability (La Menza et al., 2019). However, the abovementioned study used high rates of N fertilizer that would not be feasible in a production setting. There are few studies evaluating more practical management practices that address soybean N limitation.

Evaluating the response of soybean yield and seed composition to N fertilizer applications is key to understanding potential crop N limitations. However, this fertility practice may be unsustainable from an economic and environmental standpoint. Management practices that increase the amount of N derived from symbiotic N_2_ fixation could provide a more economically and environmentally sustainable approach. The yield response to *Bradyrhizobium japonicum* bacteria inoculations applied to the seed or during early vegetative stages depends on the field history (Albareda et al., 2009; Hungria et al., 2013; Cordeiro and Echer, 2019). Studies with previous soybean history often find no yield advantage from seed or early season inoculations (Ham et al., 1971; De Bruin et al., 2010; Carciochi et al., 2019). Interestingly, the study of Moretti et al. (2018) showed that additional spray inoculations of *B. japonicum* after planting increased nodule biomass and increased yield by 27% compared with a standard seed inoculation practice, even in a soil with a high *Bradyrhizobia* population. Symbiotic N_2_ fixation occurring in lateral roots may be inefficient because elite strains of bacteria inoculated on seeds at sowing have limited mobility and may not contribute to N_2_ fixation during seed fill (McDermott and Graham, 1989). Even though N_2_ fixation continues until the end of the soybean growing season, there are limited studies that have evaluated the effect of inoculations during early reproductive stages on soybean yield and seed composition.

Differences in the background soil inorganic N may influence the response of soybeans to the previously discussed cultural practices. A management practice of increasing popularity that could have a major impact on inorganic soil N is growing soybean following a cereal cover crop (Wells et al., 2013). Previous studies have reported mixed effects of cover crops on soybean yield, ranging from a negative effect in all or part of the sites evaluated (Eckert, 1988; Reddy, 2001; Singer and Kohler, 2005; Harasim et al., 2017; Riedell et al., 2017) to no effect on soybean yield (Ruffo et al., 2004; Uchino et al., 2009; Pantoja et al., 2015; Wen et al., 2017; Acharya et al., 2020). Some of the previous studies found a negative effect of cover crops on soybean yield that was due to an indirect reduction in population density (Eckert, 1988; Reddy, 2001; Harasim et al., 2017), which may not be associated with reduced crop N availability. The ability of the soybean crop to adapt biological N fixation to maintain yield after a cereal crop that reduces soil inorganic N remains to be further investigated. There is even less information on the effect of cover crops on soybean seed composition. A study in Iowa (Singer and Kohler, 2005) reported no effect of preceding cereal rye (*Secale cereale* L.) cover crop on soybean seed protein concentration. In contrast, a study in Poland (Harasim et al., 2017) found that soybean grown after a cereal rye or winter rape (*Brassica napus*) cover crop showed an increase in seed protein concentration, providing further evidence for soil and/or climate impacts. Further studies that evaluate the effect of cover crops on soybean yield and seed composition are needed.

In addition to seed protein concentration, meal protein concentration is a trait of interest for the soybean industry that has received little attention in agronomic studies. Meal protein concentration results from the combined effect of seed protein and oil concentration. For example, high seed oil concentrations result in relatively higher meal protein concentration after oil extraction compared with soybean seed with similar protein and lower oil concentration. In this study, we evaluated the interactive effects of a cover crop or fallow system with late-season N inputs aimed at increasing N availability during seed growth. We hypothesized that low N availability during the seed filling phase can partially limit soybean seed and meal protein concentration, which could be minimized with late-season N fertilizer applications, or with inoculant applications that may increase N_2_ fixation activity or efficiency. We also hypothesized that the response to these inputs will depend on environmental conditions, the crop yield potential, and other factors that affect soil N availability such as cover crops. To test our hypotheses we quantified the response of soybean yield and seed composition (seed oil and protein concentration, and meal protein concentration) to two late-season inputs (split application of 202 kg ha^−1^ N after R5 and *B. japonicum* inoculation at R3), under two cropping systems (soybean grown after fallow and after a cereal cover crop with residue removed), at three U.S. sites with contrasting environmental conditions (Saint Paul, Minnesota; Lexington, Kentucky; and Fayetteville, Arkansas) and using short- and full-season maturity group (MG) cultivars within each location (MG 1 and 2 in Minnesota; MG 2 and 4 in Kentucky and Arkansas).

## Materials and Methods

### Experimental Design and Treatments

Field experiments were conducted during the 2019 and 2020 soybean growing seasons at three U.S. locations between 36.03° N to 44.99° N latitude (Table 1). Soils in the experimental sites were classified as fine-silty, siliceous, active, and mesic Typic Fragiudults (Captina series) in Arkansas; fine-silty, mixed, active, and mesic Typic Paleudufals (Bluegrass-Maury series) in Kentucky; fine-silty, mixed, superactive, and mesic Typic Hapludolls (Waukegan series) in Minnesota. The experimental design was a split-split-plot randomized complete block design with four replications. The type of crop rotation (soybean after fallow or after a cover crop) was considered the main plot factor, while cultivar MG and late-season inputs (control, inoculant application at R3, and fertilizer applications after R5) were considered as the subplot and as sub-sub plot factors, respectively. A total of six cultivars were included at each location, three MG 2 cultivars and three cultivars of a MG well-adapted for a given location (MG 4 cultivars in Kentucky and Arkansas, MG 1 cultivars in Minnesota). In the N fertilizer treatment, urea was applied in two applications: 101 kg N ha^−1^ at R5, and 101 kg N ha^−1^ 2 weeks after R5. On average, the second N fertilizer application fell 5 and 10 days before soybean reached the full pod stage (R6) in short- and full-season cultivar maturities, respectively (Table 2). Liquid *B. japonicum* inoculant (Cell-Tech liquid, Novozymes BioAg, Saskatoon, Canada) was applied at R3 (or soon after, Table 2) in the inoculant treatment at a rate of 30 ml per 305 m row on the soil surface following the recommendations of the product. Inoculant was mixed with water and sprayed on the soil surface, and an irrigation event was provided in Arkansas and Kentucky after each application to help incorporate the inoculant into the soil. No measurements were taken to ensure the inoculant had effectively infected lateral soybean roots. The exact dates when inoculant and fertilizer applications were applied are summarized in Table 2.

**Table 1:** Experimental locations, latitude, and cultivars within different maturity groups (MG) used at each site.

**Location**	**Latitude**	**Soybean maturity group (MG) and cultivar**		
		**MG 1**	**MG 2**	**MG 4**
Fayetteville, AR	36.03° N	–	AG21X9 AG23X9 P21A28X	AG44X0 AG47X9 P44A72BX
Lexington, KY	38.13° N	–	AG21X9 AG23X9 P21A28X	AG44X0 AG47X9 P44A72BX
St. Paul, MN	44.99° N	CZ1139 AG14X7 AG1733	AG21X9 AG23X9 P21A28X	–

**Table 2:** Dates of field operations and soybean developmental stages at each location (Fayetteville, AR; Lexington, KY; and St. Paul, MN) and growing season, for early and full-season soybean cultivar maturity groups (MG) within each location.

**Activity and crop developmental stage**	**2019 growing season**			**2020 growing season**		
	**AR**	**KY**	**MN**	**AR**	**KY**	**MN**
Cover crop sowing^¥^	10/24/2018	10/18/2018	4/24/2019	10/28/2019	10/25/2019	10/8/2019
Cover crop termination	5/14/2019	4/29/2019	6/6/2019	4/30/2020	5/11/2020	5/26/2020
Soybean sowing	6/10/2019	6/4/2019	6/6/2019	6/2/2020	5/27/2020	5/26/2020
Inoculant (early MG)	7/24/2019	7/23/2019	8/5/2019	7/16/2020	7/20/2020	7/27/2020
Inoculant (full-season MG)	8/2/2019	7/30/2019	8/5/2019	7/28/2020	7/27/2020	8/3/2020
First dose N (early MG)	8/5/2019	8/6/2019	8/15/2019	7/28/2020	7/27/2020	8/5/2020
First dose N (full-season MG)	8/19/2019	8/14/2019	8/15/2019	8/10/2020	8/10/2020	8/5/2020
Second dose N (early MG)	8/19/2019	8/20/2019	8/29/2019	8/10/2020	8/10/2020	8/19/2020
Second dose N (full-season MG)	9/4/2019	8/28/2019	8/29/2019	8/25/2020	8/25/2020	8/19/2020
R3 date (early MG)	7/24/2019	7/22/2019	7/31/2019	7/15/2020	7/18/2020	7/20/2020
R3 date (full-season MG)	8/2/2019	7/30/2019	8/2/2019	7/27/2020	7/25/2020	7/23/2020
R5 date (early MG)	8/5/2019	8/5/2019	8/12/2019	7/31/2020	7/25/2020	8/4/2020
R5 date (full-season MG)	8/18/2019	8/12/2019	8/14/2019	8/12/2020	8/10/2020	8/9/2020
R6 date (early MG)	8/22/2019	8/20/2019	9/3/2019	8/18/2020	8/25/2020	8/24/2020
R6 date (full-season MG)	9/9/2019	9/9/2019	9/9/2019	9/5/2020	9/7/2020	9/1/2020
R7 date (early MG)	9/9/2019	9/9/2019	9/24/2019	9/5/2020	9/4/2020	9/20/2020
R7 date (full-season MG)	9/28/2019	9/24/2019	10/6/2019	9/24/2020	9/25/2020	9/24/2020

¥ * Rye was used as a cover crop in AR and KY, while oat in 2019 and a mixture of oat and wheat in 2020 were used in MN*.

### Cover Crop Establishment and Sampling

Cover crops were sown in the main plots receiving this rotation treatment. Both the fallow and cover crop main plots were tilled prior to cover crop sowing, with the exception of Kentucky, where cover crops were planted under no-till. Cereal rye was sown in Arkansas (cultivar “Elbon”) and Kentucky (cultivar “Aroostook”) the preceding fall (Table 2) at a rate of 86 and 112 kg ha^−1^, respectively. In Minnesota, due to severe cold weather and snow in the fall, an oat (*Avena sativa* L.) cover crop (cultivar “MN09103”) was planted in the spring of 2019 at a rate of 98 kg ha^−1^. In the second year of the experiment in Minnesota, winter wheat (*Triticum aestivum* L., cultivar “SY Wolf”) was sown in the fall of 2019 at a rate of 81 kg ha^−1^, and oat was direct-seeded into the wheat the following spring at a rate of 98 kg ha^−1^ (Table 2). Cover crops were terminated by mowing, baling, and removing the residue from the field in the spring. The cover crop residue was removed to minimize confounding effects of potential N additions from mineralization of the cover crop aboveground residue when testing the effect of different initial soil inorganic N availability on soybean yield and seed composition. Cover crop aboveground biomass at termination was estimated by sampling 0.25 m^2^ from four randomly selected areas (total of 1 m^2^) from each cover crop main plot. The samples were dried at 65°C until constant weight, ground, and total N concentration were determined at the Stable Isotope Laboratory at the University of Kentucky *via* combustion method using a Costech EA 4010 elemental analyzer (Costech Analytical Technologies, INC., Valencia, CA, USA).

Soil samples were taken immediately after cover crop termination from 0 to 30 cm depth by collecting 8–12 soil cores from each cover crop and fallow main plots. The samples were stored at 4°C for <2 weeks before inorganic N extractions were performed at the Agroecosystem Nutrient Cycling Laboratory at the University of Kentucky. In brief, the soil samples were homogenized by hand and a 10-g subsample was weighed fresh and after 48 h at 105°C for gravimetric moisture content determination. Ammonium- and nitrate-N were extracted from a separate 10-g fresh subsample by shaking in 40 ml of 1M potassium chloride (KCl) for 1 h. The extracts were filtered using a Whatman no. 42 filter paper. Ammonium and nitrate concentrations were determined on the filtered extracts using a colorimetric microplate method (Crutchfield and Grove, 2011). Soil inorganic N concentrations in the extracts (mg N L^−1^) were converted to units of mg N kg^−1^ dry soil using the volume of KCl added, the soil mass extracted, and soil moisture content. Soil inorganic N concentration (mg kg^−1^) was expressed as the total amount of N in the top 30 cm of soil (kg N ha^−1^) using measured bulk density in Kentucky (1.33 g cm^−3^) and with bulk density estimated from the Web Soil Survey for the sites in Minnesota (1.45 g cm^−3^) and Arkansas (1.40 g cm^−3^).

### Soybean Management and Data Collection

Soybean was seeded at a rate of 35 seeds m^−2^ in both fallow and cover crop subplots on the day of cover crop termination or up to 36 days later (Table 2). Fields were tilled before planting, and P and K fertilizer were applied depending on the year and location according to soil tests and best fertility management recommendations for soybean from extension guides at each site. Soybean seeds were treated with *B. japonicum* commercial powdered peat or liquid inoculant (Advanced Biological Marketing, Van Wert, Ohio, USA) before planting. Plots in Arkansas consisted of 4 rows which were 46 cm apart and 7.5 m long. In Kentucky, plots had 6 rows which were 38 cm apart and 7.5 m long. Plots in Minnesota consisted of 4 rows which were 76 cm apart and 4.6 m long in 2019, and 7.5 m long in 2020. Pre-emergent herbicide was applied immediately after sowing for weed control and post-emergent herbicides were applied during the growing season as needed. Experiments in Minnesota were rainfed, while soybean was irrigated in Arkansas and Kentucky using a sprinkler and a drip-tape irrigation system, respectively. Irrigation was applied when the cumulative soil water deficit reached 40 mm, as determined using a daily water balance of precipitation and the crop evapotranspiration demand, and utilizing the FAO-56 methodology with a dual crop-coefficient approach as described by the study of Purcell et al. (2007).

Soybean growth and development were monitored to record the date of the beginning pod (R3), beginning seed (R5), full seed (R6), physiological maturity (R7), and harvest maturity (R8) as described by the study of Fehr and Caviness (1977). Dates of R3, R5, and R6 were recorded in control plots from two replicates to identify the time to apply inoculant and N fertilizer applications. Dates of R7 and R8 were recorded from all plots to analyze treatment effects. Node number was recorded at R7 in all plots. Soybean yield was determined by harvesting two central rows, each having 5.49 m length in Arkansas (3.3 m^2^). In Kentucky, four central rows of 3.05 m length were harvested (4.6 m^2^). In Minnesota, two central rows of 1 m length were harvested in 2019 (1.5 m^2^), and a length of 5.5 m was harvested in 2020 (8.4 m^2^). Seed yield was adjusted to a moisture content of 130 g kg^−1^. There were 100 seed weights recorded from three subsamples of harvested seed from each plot to obtain the average individual seed weight, which was then used to quantify the number of seeds per area.

Seed protein and oil concentrations were analyzed in whole seed harvest samples from each plot using a DA 7250™ near-infrared spectroscopy (NIRS) analyzer (Perten Instruments, Hägersten, Sweden) fitted with equations developed by the University of Minnesota. The expected meal protein concentration following the extraction of oil from whole seed samples was estimated based on the model from the study by Updaw et al. (1976) and following Equation 1 provided by the study of Brumm and Hurburg (1990). This equation assumes soybean is processed with a moisture content of 130 mg g^−1^, a test weight of 772 g L^−1^ (60 lb bu^−1^), a 1.15% total dry matter loss in the crushing process, a residual oil content in the meal of 12 mg g^−1^, and that the resulting meal has moisture of 120 mg g^−1^.

*Meal protein concentration (mg g*^−1^*)* = −*1.343* + *0.6712 Oil* + *1.3203 Protein* (1)

Where *Oil* and *Protein* are concentration in mg g^−1^ of oil and protein in whole seeds expressed on a 130 mg g^−1^ moisture basis, respectively.

### Statistical Analysis

Soybean data were analyzed with an ANOVA using Proc Glimmix in SAS v 9.4 (SAS Institute, Cary, North Carolina, USA). Year, location, rotation type, late-season input treatment, cultivar MG nested within the location, cultivar nested within MG and location, and their interactions were considered as fixed factors in the model. To simplify field operations of cover crop planting and termination, the rotation type was randomized within two sections in the field (lots) and each lot was then split into two blocks. Lot, which was nested within year and location, and block, which was nested within lot, and their interactions with other fixed effects were considered as random factors. The effect of rotation type and late-season input treatment on the variables measured was analyzed by generating the least significant differences for the highest level interaction with these fixed effects that was significant at *P* < 0.05. To visualize measured variables averaged across similar factors when an interaction level was not significant, we provided means by location, year, rotation type (cover crop or fallow), late-season input treatment (control, inoculant application at R3, and N fertilizer applications after R5), and averaged across cultivars in the Supplementary Figures 1–3.

The relationship between meal protein concentration and soybean yield was analyzed with a quadratic model using the Proc Reg procedure in SAS. The quadratic term was dropped from the model when not significant at a probability of 0.05. A model fit was obtained for each rotation type and late-season input treatment combining data across all years and locations.

## Results

### Cover Crop Biomass and Residual Soil Inorganic N

Cover crop aboveground biomass was not statistically compared across locations or years, but it was numerically highest each year at the southernmost location in the study and lowest in the northernmost location where cover crops were sown in the spring (Table 3). Cover crop aboveground N content at termination did not always follow a similar pattern to aboveground biomass and ranged from 14 to 45 kg ha^−1^ (Table 3). Soil inorganic N in the top 30 cm after the cover crop termination did not differ by more than 10 kg N ha^−1^ in most cases between the fallow and cover crop treatments. One exception was the site in Arkansas in 2019, where soil inorganic N was 22 kg ha^−1^ less in the cover crop treatment compared with the fallow (Table 3). Overall, the reduction in soil inorganic N in cover crop treatments compared with fallow was always lower compared with the amount of N content in cover crop biomass (Table 3).

**Table 3:** Average (+/– SE) cover crop biomass, aboveground N content, and soil inorganic N in the top 0.3 m at cover crop termination.

	**2019**			**2020**		
	**AR**	**KY**	**MN**	**AR**	**KY**	**MN**
Cover crop biomass (kg/ha)	5,110 ± 321	3,875 ± 224	588 ± 61	6,353 ± 419	3,059 ± 337	1,578 ± 150
Cover crop N content (kg/ha)	34.7 ± 4.2	42.2 ± 3.9	13.6 ± 2.0	64.7 ± 6.0	30.8 ± 2.0	44.6 ± 4.5
Soil inorganic N (kg/ha) after fallow	58.5 ± 0.2	22.5 ± 1.0	19.8 ± 0.6	12.8 ± 2.4	4.4 ± 4.4	11.1 ± 3.5
Soil inorganic N (kg/ha) after cover crop	36.3 ± 2.2	16.8 ± 2.6	10.5 ± 1.9	4.9 ± 4.4	7.8 ± 3.5	6.8 ± 1.0

### Environmental Conditions During Soybean Growing Season

The daily mean temperature during soybean vegetative (EV–R1) and early reproductive (R1–R5) phases differed by 3.5 and 4.7°C on average across locations, respectively (Table 4). During the late reproductive phase or seed-fill (R5—R7), differences across the three sites in mean daily temperature increased to 6.9°C on average across locations. Daily solar radiation intensity also showed the largest differences across locations during the late reproductive phase, ranging from 16.6 MJ m^−2^ day^−1^ at the site in Minnesota to 19.4 MJ m^−2^ day^−1^ at the site in Arkansas. Total precipitation during the soybean growing season ranged from 267 to 505 mm depending on the location and year (Table 4) and was supplemented with irrigation with the exception of the site in Minnesota.

**Table 4:** Mean daily temperature, solar radiation intensity, and total precipitation during vegetative (VE–R1), early reproductive (R1–R5), and late reproductive (R5–R7) phases in soybean trials grown at three locations during 2019 and 2020.

**Location and year**	**Mean daily temperature (** ^**°**^ **C)**			**Mean daily solar radiation intensity (MJ m**^**−1**^ **day**^**−1**^**)**			**Total precipitation (mm)**		
	**VE–R1**	**R1–R5**	**R5–R7**	**VE–R1**	**R1–R5**	**R5–R7**	**VE–R1**	**R1–R5**	**R5–R7**
**Fayetteville, AR**									
2019	25.6	25.9	26.1	23.9	22.7	18.9	114	89	213
2020	25.2	26.7	24.2	23.9	21.7	19.9	92	185	81
**Lexington, KY**									
2019	24.5	25.6	25.0	22.3	23.5	19.9	143	63	159
2020	23.0	25.9	23.3	23.9	23.4	18.6	108	34	160
**St. Paul, MN**									
2019	21.8	21.4	17.9	19.3	21.6	15.5	146	63	297
2020	22.1	21.9	18.7	23.6	22.6	17.7	99	52	116

### Soybean Development, Yield, and Yield Components

**Figure 1:**
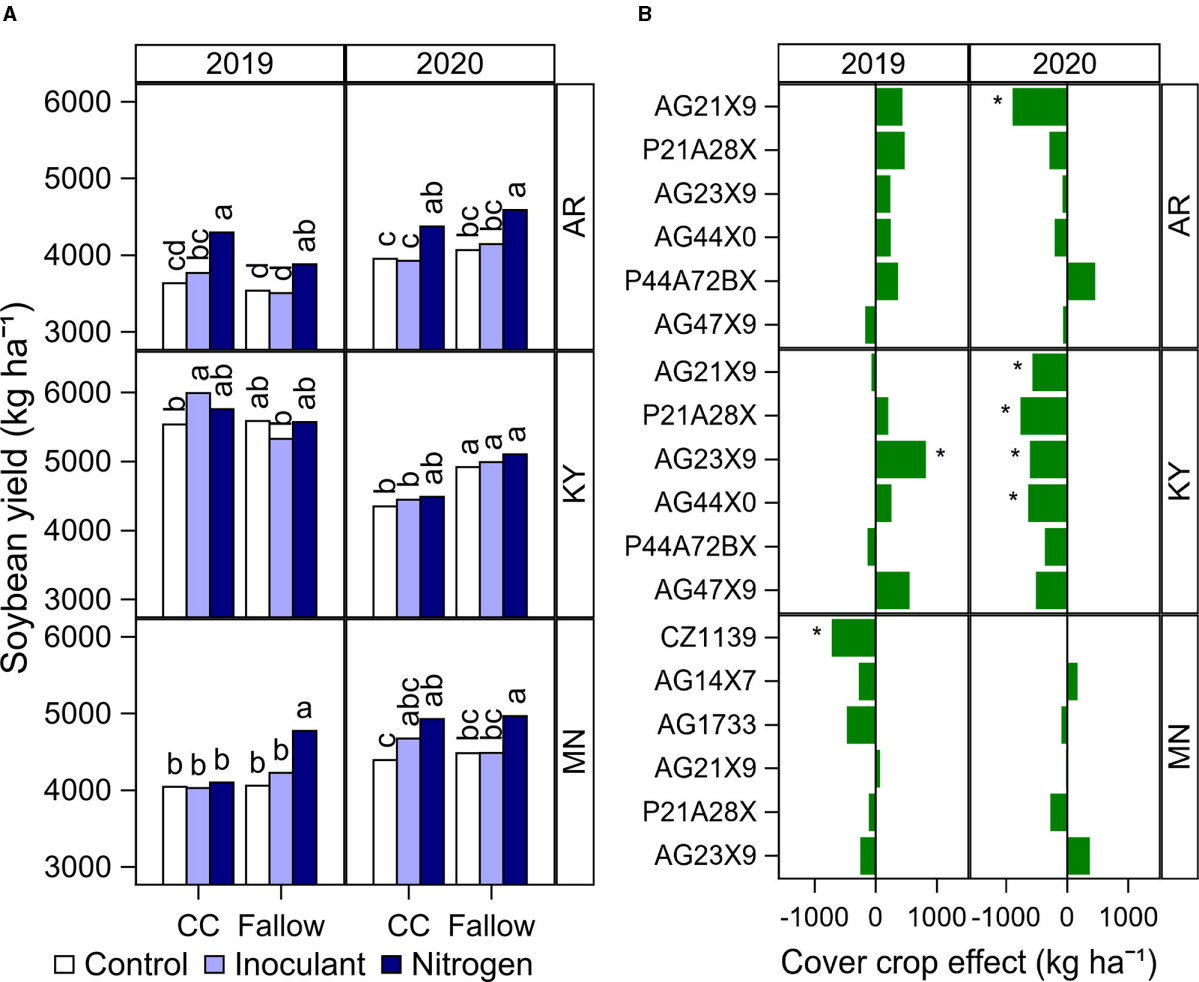
Summary of significant effects of cultural practices on soybean yield. **(A)** Soybean yield by rotation type (CC, cover crop; or fallow) and late-season input treatment (Control, Inoculant application at R3, and N fertilizer applications after R5) at each location and year. Data averaged across cultivars. Different letters above bars within a year and location indicate significantly different means at *P* < 0.05. **(B)** The net effect of cover crop (cover crop–fallow) on soybean yield by cultivar at each location and year. The asterisk indicates a significant cover crop effect at *P* < 0.05.

**Figure 2:**
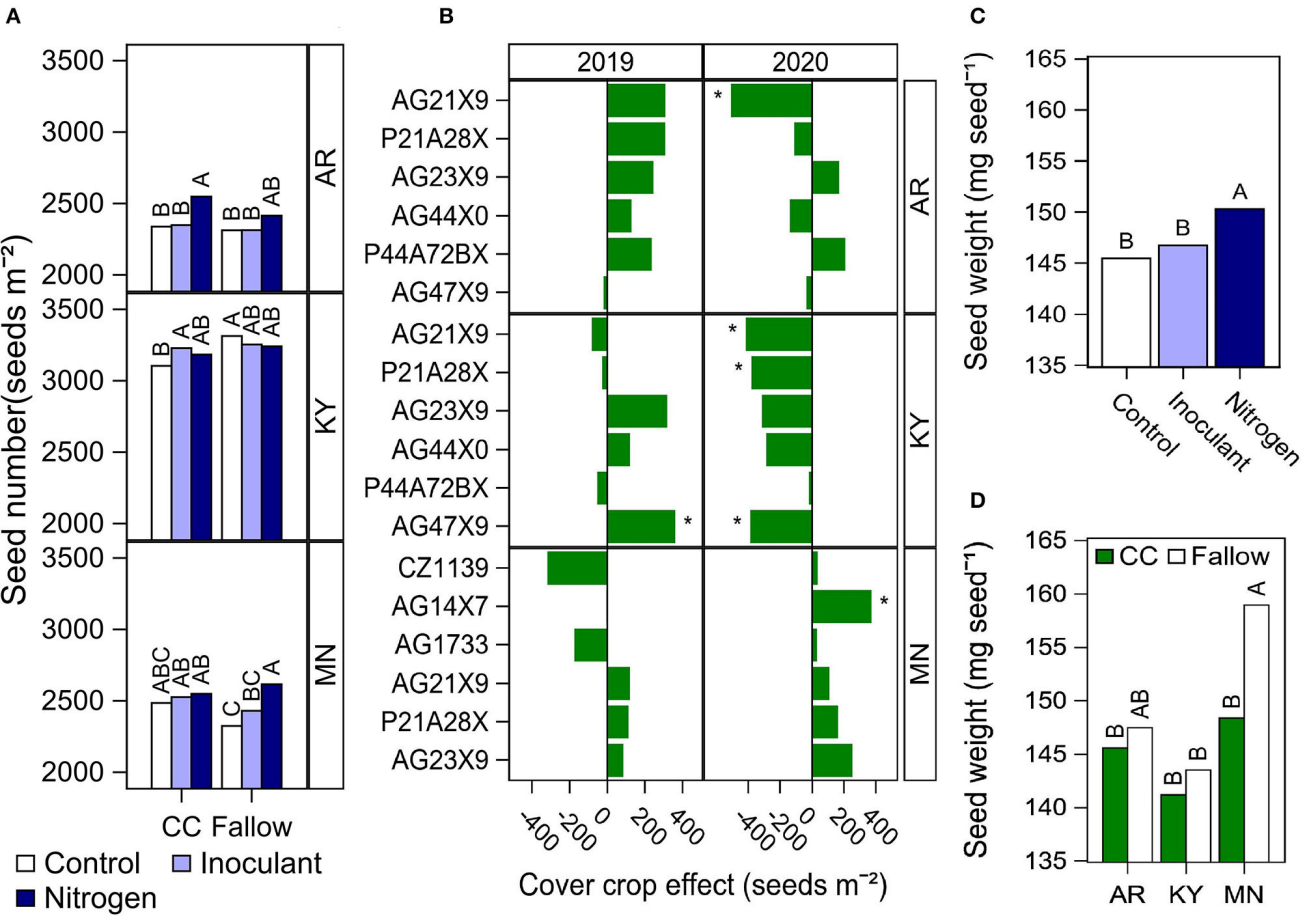
Summary of significant effects of cultural practices on soybean yield components. **(A)** Soybean seed number by rotation type (CC, cover crop; or fallow) and late-season input treatment (Control, Inoculant application at R3, and N fertilizer applications after R5) at each location. Data averaged across cultivars and years. **(B)** Effect of cover crop (Fallow–cover crop) on soybean seed number by cultivar at each location and year. The asterisk indicates a significant cover crop effect at *P* < 0.05. **(C)** Soybean seed weight by late-season input treatment (Control, Inoculant application at R3, and N fertilizer applications after R5). Data averaged across locations, years, rotation types, and cultivars. **(D)** Soybean seed weight by rotation type (CC, cover crop; or fallow) at each location. Data averaged across years, late-season input treatments, and cultivars. Different letters above each bar indicate significantly different means at *P* < 0.05.

**Figure 3:**
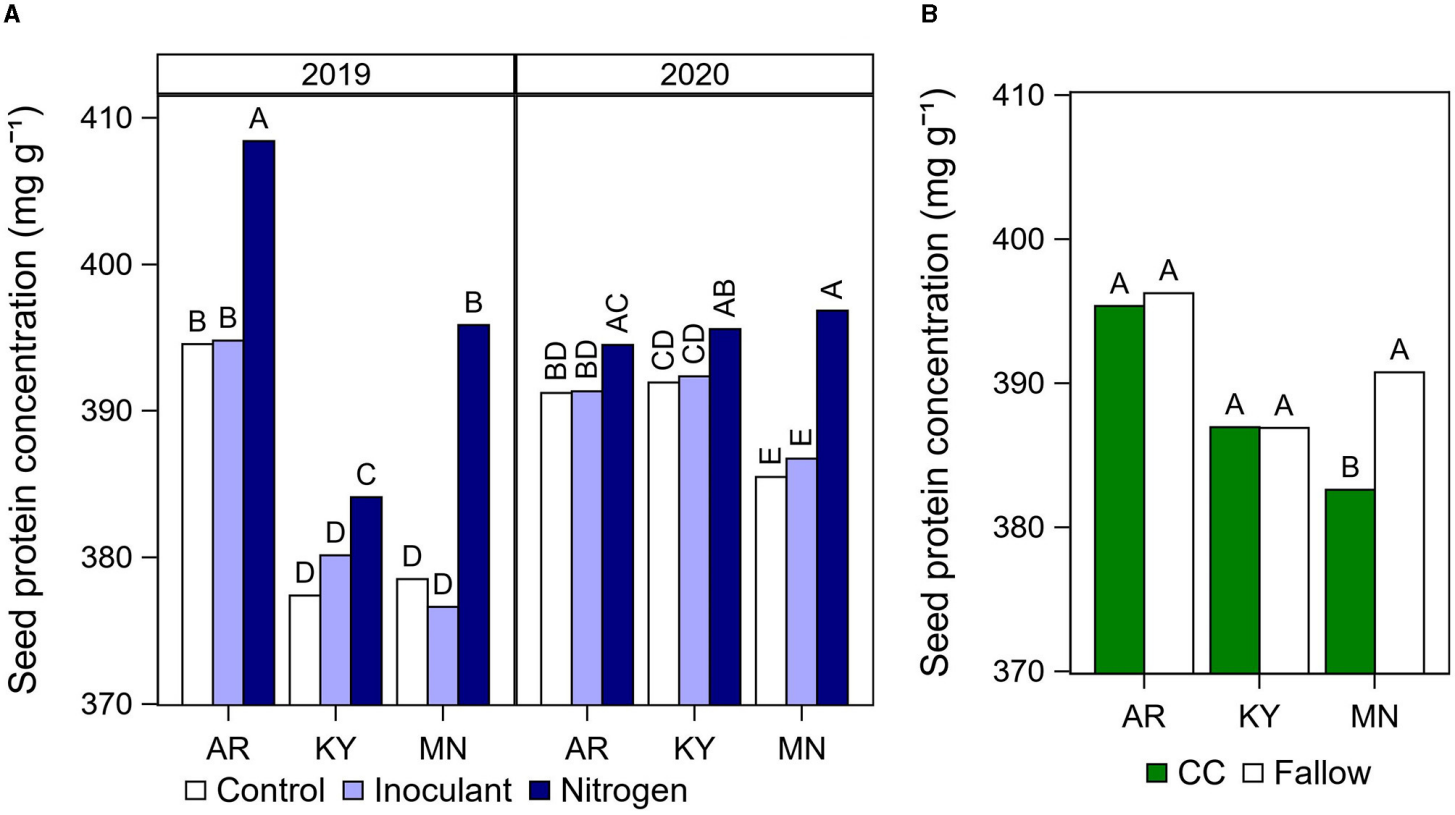
Summary of significant effects of cultural practices on seed protein concentration. **(A)** Soybean seed protein concentration by late-season input treatment (Control, Inoculant application at R3, and N fertilizer applications after R5) at each location and year. Data averaged across rotation types and cultivars. **(B)** Seed protein concentration by rotation type (CC, cover crop; or fallow) at each location. Data averaged across years, late-season input treatments, and cultivars. Different letters above each bar indicate significantly different means within a year and location at *P* < 0.05.

**Figure 4:**
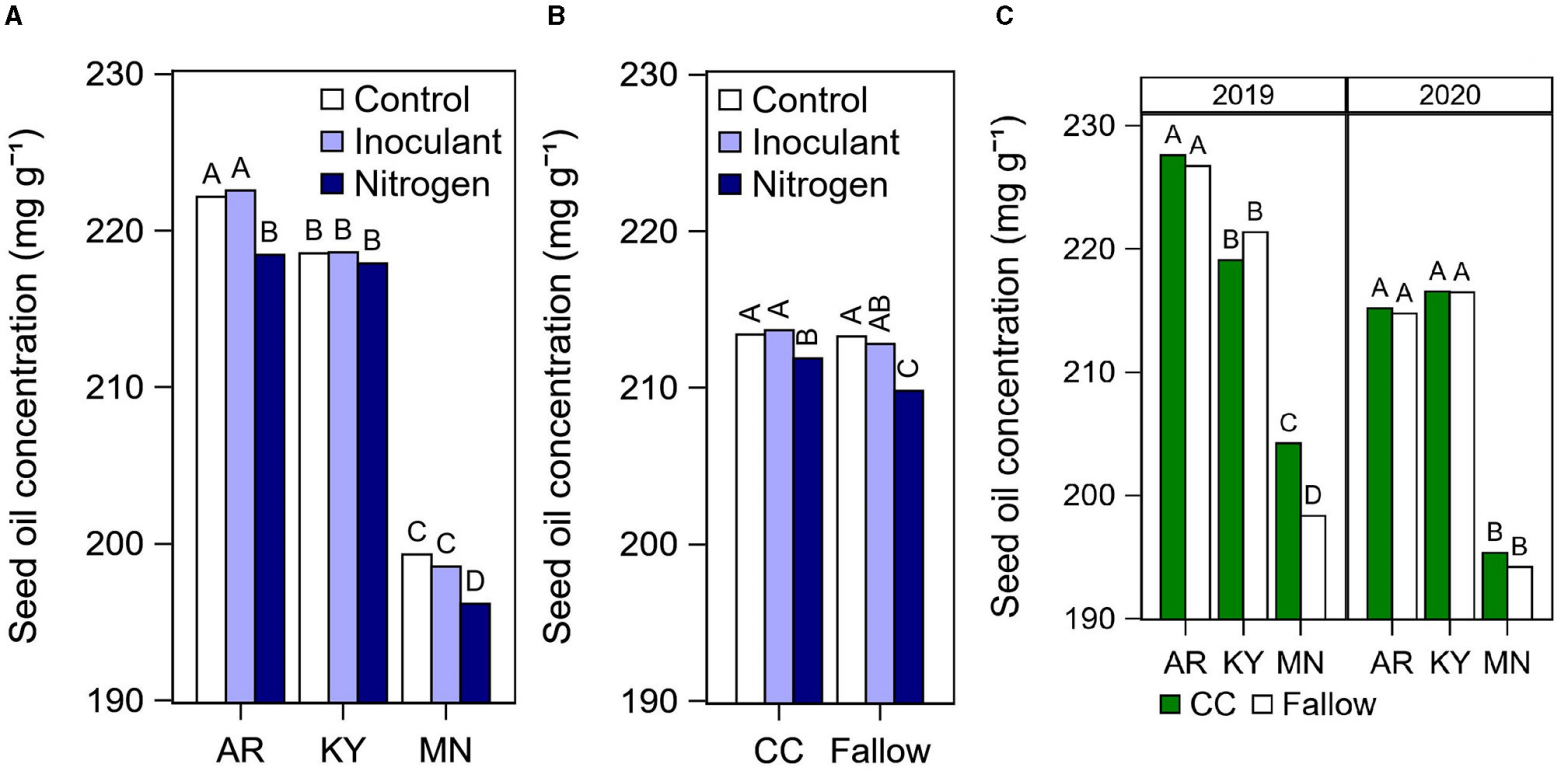
Summary of significant effects of cultural practices on seed oil concentration. **(A)** Soybean seed oil concentration by late-season input treatment (Control, Inoculant application at R3, and N fertilizer applications after R5) at each location. Data averaged across years, rotation types, and cultivars. **(B)** Seed oil concentration by rotation type (CC, cover crop; or fallow) and late-season input treatment. Data averaged across years and cultivars. **(C)** Soybean seed oil concentration by rotation type at each location and year. Data averaged across late-season input treatments and cultivars. Different letters above bars within a year and location indicate significantly different means at *P* < 0.05.

**Figure 5:**
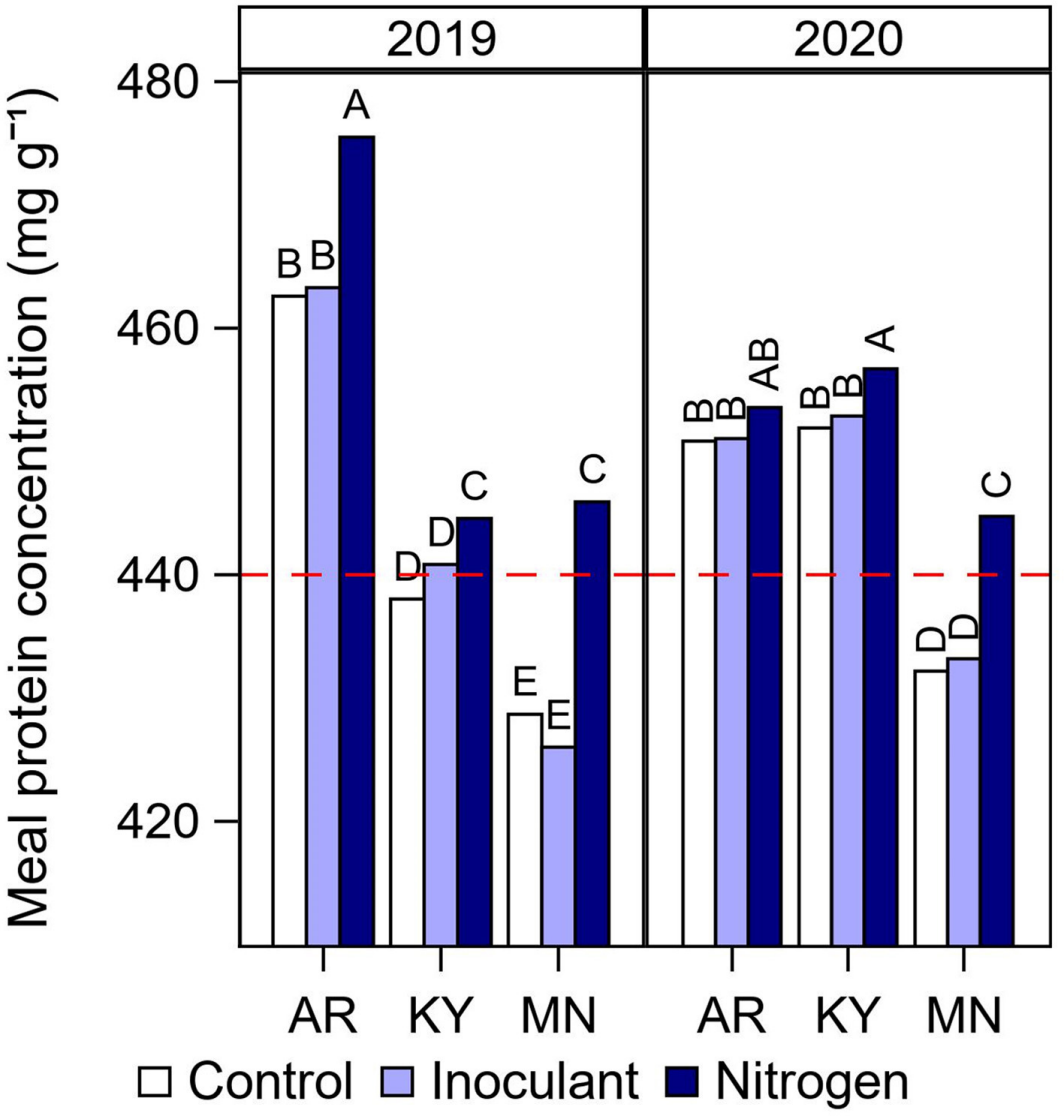
Summary of interactive effects of cultural practices on meal protein concentration. Meal protein concentration by late-season input treatment (Control, Inoculant application at R3, and N fertilizer applications after R5) at each year and location. Data averaged across rotation types and cultivars. Different letters above bars within a year indicate significantly different means at *P* < 0.05. The red dashed line indicates the minimum protein concentration threshold in non-dehulled soybean meal required by the processing industry for high protein meal designation.

Analysis of variance showed significant effects of the rotation and late-season input treatments but they were dependent on the location, year, or cultivar depending on the variable analyzed (Table 5). Thus, results were summarized in Figures that show the highest level interaction that was significant at *P* < 0.05 for the evaluated treatments and rotation effects (Figures 1–5). For instance, soybean yield was affected by the interaction between rotation type and treatment depending on the year and location (Table 5, Figure 1A). In addition, rotation affected soybean yield depending on the cultivar at each year and location (Table 5, Figure 1B). The N fertilizer application had a positive effect on soybean yield without cover crops in two out of three locations (Figure 1A). The N fertilizer increased soybean yield in Arkansas by 13% on average across cultivars compared with the unfertilized control. In Minnesota, N fertilizer increased soybean yield by 18% on average across cultivars for the fallow treatment in 2019, and by 11% in 2020 compared with the control. In Kentucky, the yield was not significantly different between the control and N treatment in either year, but the crop rotation decreased yields by 13% on average relative to the fallow in 2020 (Figure 1A). The inoculant treatment did not affect soybean yield, except for 2019 in Kentucky where it increased yield by 12% on average across cultivars under the cover crop rotation compared with the fallow (Figure 1A). Growing soybean after a cover crop had a negative effect on yield in 6 out of 36 cultivars by year and location combinations (11–22% yield decrease relative to the fallow), and a positive effect in one occasion (17% yield increase) (Figure 1B). As expected, yields were 4–31% higher in the full-season cultivars compared with the relatively short-season cultivars within each location (data not shown). However, we did not find an interactive effect of the treatments evaluated with the cultivar MG (Table 5).

**Table 5:** Probabilities from the ANOVA analysis of yield, seed number, seed weight, node number, date of physiological maturity (R7), harvest maturity (R8), seed oil and protein concentrations, and meal protein concentration.

**Effect**	**DF^¥^**	**Yield (kg ha^**−1**^)**	**Seed number (seeds ha^**−1**^)**	**Seed weight (mg seed^**−1**^)**	**Main stem node number**	**Date of R7**	**Date of R8**	**Seed oil (mg g^**−1**^)**	**Seed protein (mg g^**−1**^)**	**Meal protein (mg g^**−1**^)**
Year (Y)	1	0.9157	0.0075	0.0559	0.5003	<0.0001	0.0002	0.0004	0.0106	0.8910
Location (L)	2	0.0044	0.0001	0.1522	0.0198	<0.0001	<0.0001	<0.0001	0.0007	<0.0001
Y*L	2	0.0338	0.0162	0.2995	0.2187	0.0013	0.0089	0.0391	0.0009	0.0003
Rotation (R)	1	0.3031	0.8201	0.0115	0.2426	0.0009	0.0071	0.0468	0.0311	0.0607
Y*R	1	0.0761	0.1143	0.3463	0.9466	0.0905	0.6484	0.2864	0.6961	0.8622
L*R	2	0.5463	0.2944	0.0701	0.9108	0.0005	0.0072	0.0103	0.0386	0.1092
Y*L*R	2	0.0512	0.0719	0.5491	0.8540	0.0717	0.5779	0.0332	0.2251	0.4827
Treatment (T)	2	<0.0001	<0.0001	0.0127	0.4759	0.0014	<0.0001	<0.0001	<0.0001	<0.0001
Y*T	2	0.8427	0.1719	0.4237	0.6618	0.1486	0.0255	<0.0001	<0.0001	<0.0001
L*T	4	0.0022	0.0150	0.7997	0.7167	0.1482	0.0007	0.0005	<0.0001	<0.0001
Y*L*T	4	0.9695	0.9067	0.7345	0.7262	0.7475	0.0014	0.5536	0.0098	0.0103
R*T	2	0.1113	0.6922	0.2282	0.1496	0.4699	0.4252	0.0212	0.2564	0.5026
Y*R*T	2	0.5785	0.4878	0.5777	0.3044	0.8937	0.9708	0.3255	0.0670	0.0556
L*R*T	4	0.1158	0.0151	0.4966	0.1429	0.7942	0.0657	0.9509	0.8999	0.8835
Y*L*R*T	4	0.0357	0.4544	0.5787	0.2939	0.6817	0.0975	0.4223	0.8242	0.8684
MG(L)	3	<0.0001	<0.0001	0.0103	0.0360	<0.0001	<0.0001	<0.0001	<0.0001	<0.0001
Y*MG(L)	3	<0.0001	0.1233	<0.0001	0.0860	<0.0001	<0.0001	0.0010	<0.0001	<0.0001
R*MG(L)	3	0.7309	0.4016	0.4199	0.8814	0.5759	0.7906	0.9152	0.2295	0.1034
Y*R*MG(L)	3	0.2491	0.2926	0.9944	0.8826	0.8796	0.1434	0.8566	0.6386	0.4233
T*MG(L)	6	0.9153	0.6023	0.8788	0.6468	0.9199	0.3432	0.1123	0.8694	0.9177
Y*T*MG(L)	6	0.2552	0.6602	0.8153	0.7788	0.1471	0.6237	0.7905	0.2151	0.2453
R*T*MG(L)	6	0.6776	0.8919	0.9565	0.1776	0.8229	0.4253	0.8094	0.6118	0.4561
Y*R*T*MG(L)	6	0.3862	0.2118	0.8045	0.2523	0.7099	0.4558	0.3549	0.5839	0.6906
Cultivar(L*MG)	12	<0.0001	<0.0001	<0.0001	0.7362	<0.0001	<0.0001	<0.0001	<0.0001	<0.0001
Y*Cultivar(L*MG)	12	<0.0001	0.0002	0.7658	0.7705	<0.0001	<0.0001	<0.0001	<0.0001	<0.0001
R*Cultivar(L*MG)	12	0.0255	0.0271	0.1843	0.1264	0.1018	0.0484	0.2148	0.1360	0.2542
Y*R*Cultivar(L*MG)	12	0.0149	0.0348	0.2674	0.1495	0.9723	0.2745	0.5529	0.2607	0.2533
T*Cultivar(L*MG)	24	0.8672	0.9721	0.9789	0.2480	0.0136	0.1308	0.7412	0.0560	0.2429
Y*T*Cultivar(L*MG)	24	0.6477	0.7554	0.9939	0.2131	0.0041	0.8749	0.5537	0.0985	0.3394
R*T*Cultivar(L*MG)	24	0.5581	0.3492	0.1020	0.8588	0.1666	0.8668	0.8617	0.4326	0.7053
Y*R*T*Cultivar(L*MG)	24	0.2057	0.5454	0.1229	0.7752	0.7472	0.9847	0.6925	0.4746	0.3272

¥ *DF, Degrees of freedom*.

The yield increase in Arkansas and Minnesota for the N fertilizer treatment was due to a higher seed number depending on the rotation type (Figure 2A) and by a 3% increase in average final seed weight (Figure 2C) compared with the control. The positive or negative effect of cover crop on soybean yield (Figure 1B) was explained primarily by its effect on seed number (Figure 2B) and partially explained by a reduction in seed weight in Minnesota (Figure 2D). The cultural practices evaluated did not affect the total node number in our indeterminate cultivars (Table 5). The date of R7 was affected by the late-season treatments, but with a negligible delay of <1 day in the N fertilizer treatment compared with the control (Supplementary Figure 4A). The cover crop delayed the date of R7 in Minnesota by 4 days (Supplementary Figure 4B). This delay did not seem associated with a delay in the date of emergence or early developmental stages since the dates of R1 and R5 only differed by 1 day on average between cover crop and fallow treatments in Minnesota (data not shown). The N fertilizer treatment delayed the date of R8 in Arkansas (2019) and Minnesota (both years), but only by 1 or 2 days (Supplementary Figure 4C).

### Soybean Seed and Meal Composition

The N fertilizer treatment increased seed protein concentration compared with the control and the inoculant treatments in all cases, with the exception of Arkansas in 2020 where this effect was not significant (Figure 3A). On average across both years at each location, the N fertilizer application after R5 increased protein concentration by 13 mg g^−1^ (Arkansas), 5–7 mg g^−1^ (Kentucky), and 13–20 mg g^−1^ (Minnesota) relative to the unfertilized control. In Minnesota, growing soybean after a cover crop decreased seed protein concentration by 8 mg g^−1^ on average (Figure 3B), but increased oil by 6 mg g^−1^ in 2019 (Figure 4C) compared with the fallow.

The N fertilizer applications decreased seed oil concentration by 4 mg g^−1^ in Arkansas and 3 mg g^−1^ in Minnesota relative to the control (Figure 4A). However, the total oil yield in kg ha^−1^ was still the highest under the N fertilizer treatment due to the increase in yield in this treatment (Supplementary Figure 5). Growing soybean after a cover crop lessened the negative effect of N fertilization on oil concentration by 2 mg g^−1^ on average across all locations compared with the fertilized fallow (Figure 4B).

**Figure 6:**
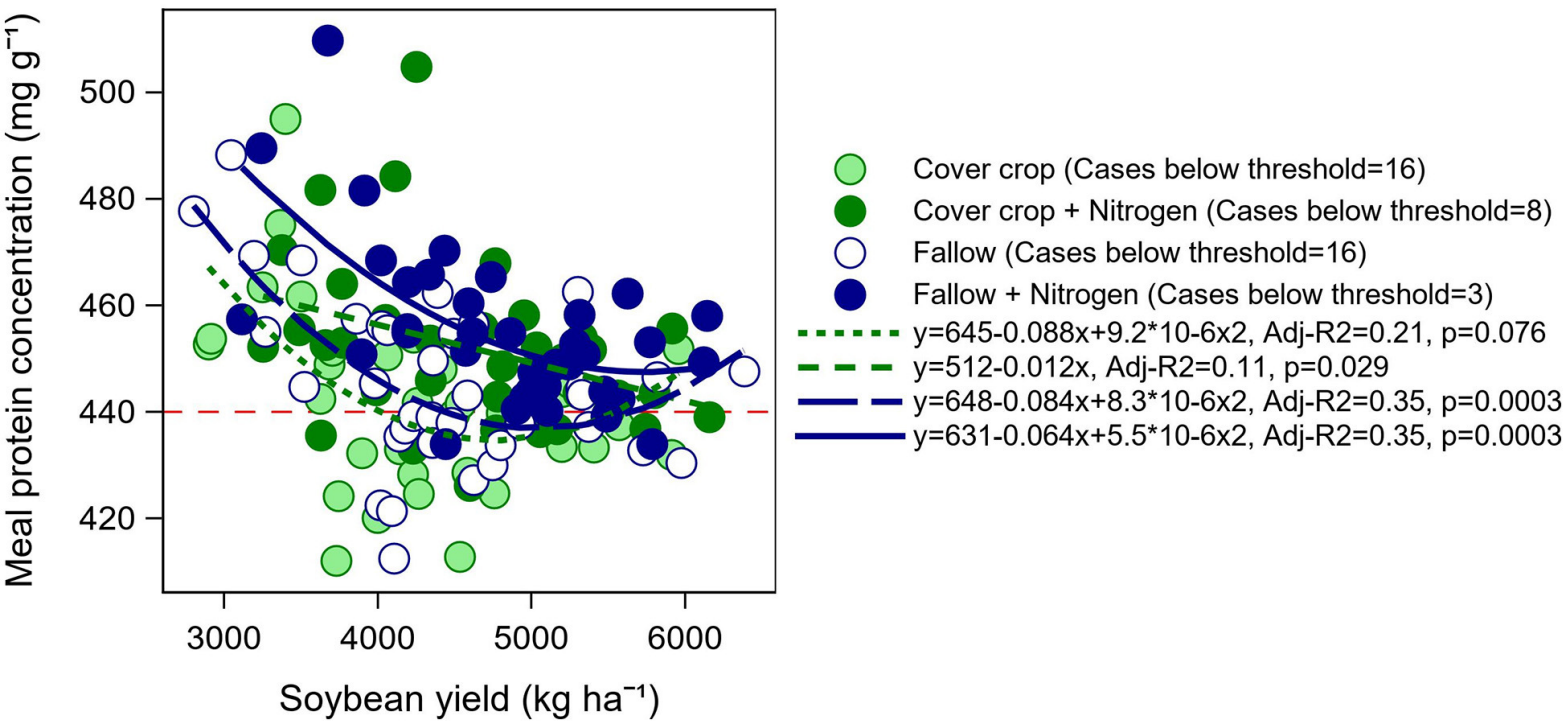
Relationship between soybean yield and meal protein concentration by late-season input treatment and rotation type. Data are shown for each cultivar, location, and year. The horizontal red dashed line indicates the minimum protein concentration threshold in non-dehulled soybean meals required by the processing industry for high protein meal designation.

Average meal protein concentration by year, location, and late-season input treatment (Figure 5) followed a similar pattern to that observed for seed protein concentration (Figure 3A). As expected, meal protein concentration was the lowest in Minnesota and fell below the minimum threshold required by the industry of 440 mg g^−1^ for high meal protein designation (Figure 5). However, it is interesting to note that although seed protein concentration was similar in Kentucky and Minnesota in 2019 (Figure 3A), meal protein concentration was the lowest in Minnesota due to the relatively lower seed oil concentration at this northernmost location (Figure 4A). Of additional interest, meal protein concentration fell below the minimum required threshold in Kentucky 2019 which was coincident with the highest yields recorded in our study (5,630 kg ha^−1^ on average, Figure 1A). The N fertilizer treatment increased meal protein concentration in Kentucky and Minnesota above the 440 mg g^−1^ threshold (Figure 5). For individual cultivars, meal protein concentration fell below the minimum threshold in 32 out of 72 cases under the control treatment, i.e., 16 cases under both soybeans after fallow and after a cover crop (Figure 6). Most of these cases occurred in Minnesota (20), followed by Kentucky (8), and Arkansas (4) (Supplementary Figure 6). The N fertilizer treatment reduced the number of cases when meal protein concentration fell below the minimum threshold to three cultivars under fallow, and eight cultivars after a cover crop (Figure 6). The relationship between meal protein concentration and yield was best described by a negative asymptotic model (Figure 6). Hence, we observed a rapid decline in meal protein concentration with increasing yield, but meal protein concentration had a tendency to decrease to a lesser extent or remain constant with yields above 5,000 kg ha^−1^. Both the N fertilizer treatment and the type of rotation affected the shape of this relationship to some extent.

## Discussion

### Effect of N Fertilizer Applications After R5

There is a pressing need to develop management solutions that halt the decreasing trend in soybean seed protein concentration. We tested the hypothesis that cultural practices which increase N availability during seed fill could have an impact on seed composition using a high rate of N fertilizer applied after R5 (202 kg N ha^−1^). We found that N fertilizer applications after R5 increased seed protein concentration by 5–20 mg g^−1^ relative to the unfertilized control across multiple cultivars, sites, and years. Interestingly, the increase in seed protein concentration with N fertilizer applications was the greatest at our northernmost site in Minnesota, i.e., 13–20 mg g^−1^ increase relative to the unfertilized control. The low seed protein concentration is most concerning in northern U.S. states, which typically have a lower protein concentration compared with production areas in the south (Hurburgh Jr et al., 1990; Rotundo et al., 2016). The late N fertilizer treatment increased seed protein concentration in Minnesota to values similar to those obtained without N fertilizer applications in Arkansas, and above the seed protein concentration measured in Kentucky (Figure 3). In addition, N fertilizer applications increased yield in Minnesota and Arkansas by 11–13% or 1,789–1,950 kg ha^−1^, which from an economic standpoint could justify applications of N fertilizer to increase seed protein concentration in some areas.

Results from this study indicated that there is a potential to address the declining trend in seed protein concentration with management-based solutions. A previous study conducted by Ham et al. (1975) in Minnesota which applied 224 kg N ha^−1^ at planting found an increase in seed protein concentration by 2–6 mg g^−1^ in 4 out of 5 site years, but in low yielding conditions (average yield was 1,822 kg ha^−1^ and ranged from 1,042 to 2,862 kg ha^−1^ across sites and nodulating cultivars used in the study). Subsequent studies found an increase in seed protein concentration evaluating very high rates of N fertilizer (Wilson et al., 2014; La Menza et al., 2017, 2019). The study by Wilson et al. (2014) evaluated cultivars released from 1923 to 2008 across locations in the U.S. Midwest with N fertilizer applications of 560 kg N ha^−1^ split between planting and found an increase in seed protein concentration in MG 2 cultivars but not in yield. The N fertilizer applications increased yield but not seed protein concentration of MG 3 cultivars in the same study (Wilson et al., 2014). The relatively early- and full-season MG cultivars evaluated in our study had a main effect on yield and seed composition but did not differ in their response to the cultural practices evaluated. The study of La Menza et al. (2019) conducted experiments in Balcarce, Argentina, and Nebraska, USA, with high rates of N fertilization (540–870 kg N ha^−1^) split into several applications between V2 to R5 stages and found an average of 11% (0.46 Mg ha^−1^) increase in yield and a 15 mg g^−1^ increase in seed protein concentration. Our results indicated that relatively lower rates of N fertilizer applied during seed growth may be as effective to increase yield and seed protein concentration as high rates of N fertilizer applied throughout the growing season in non-water stressed soybean.

In contrast, other studies with N fertilizer applications from R1 to R4 found no effect on seed protein concentration (Wesley et al., 1998; Gutiérrez-Boem et al., 2004; Ortez et al., 2018). The study of Wesley et al. (1998) evaluated low rates of 22 and 44 kg N ha^−1^ applied at R3 with no effect on seed protein, although the yield was improved in six out of eight locations in the study. In the study conducted by Ortez et al. (2018), they evaluated N fertilizer applications as high as 670 kg ha^−1^ (equally split at planting, R1, and R3–R4) with no effect on seed protein concentration. The study of Gutiérrez-Boem et al. (2004) found no effect on soybean seed protein concentration and yield with 50 or 100 kg N ha^−1^ applied at either R3 or R5 stages. It is possible that our second split-application of N fertilizer 2 weeks after R5 has contributed to increasing seed protein concentration, which partially explains the different results from the study by Gutiérrez-Boem et al. (2004). Lastly, previous studies evaluating early N applications at planting and up to the R1 growth stage found no effect (Osborne and Riedell, 2006; Wilson et al., 2014) and even reductions (Ray et al., 2006; Kaur et al., 2017) in seed protein concentration. N fertilizer applications were found to decrease protein in irrigated trials in Mississippi (Ray et al., 2006; Kaur et al., 2017). Early season N applications decrease root nodulation and consequently biological N fixation (Gan et al., 2002; Salvagiotti et al., 2008; Cordeiro and Echer, 2019). Thus, early-season N applications could have an undesirable effect on soybean yield and seed protein concentration by reducing nodule mass and the ability to fulfill high rates of N accumulation to the seeds during seed fill.

The late N fertilizer applications increased yield in two out of three locations due to an increase in seed number depending on the location and year, and also an increase in seed weight (Figures 2A,C). The window of flowering and pod addition in soybean is mostly completed by the R5 stage. Other studies found an increase in seed number with N fertilizer applications before or during the period of pod setting (Purcell and King, 1996; La Menza et al., 2017; Cordeiro and Echer, 2019). The increase in seed number that we found with N fertilizer applications after R5 was unexpected and could be explained by a reduction in flower and pod abortion during the end of the flowering and pod setting window. The cultivars in our study were all indeterminate but we did not find that N fertilizer applications increased seed number through node addition (Table 5). Other studies found an increase in seed weight in response to N fertilizer applications while evaluating high rates of 540 kg N ha^−1^ or more (Wilson et al., 2014; La Menza et al., 2017; Ortez et al., 2018). Increases in seed weight in response to N fertilizer applications might be less common compared with increases in seed number given that seed number is determined first and there is a downregulating compensatory mechanism between these two yield components. The N fertilizer applications delayed the date of R7 and R8 by <2 days compared with the unfertilized control, suggesting that the increase in seed weight was partially due to an increase in the rate of seed growth.

Overall, the N fertilizer applications during the period of seed growth were effective in increasing both seed protein concentration and yield in most cases, and in particular at our northernmost location where low seed protein is a concern. However, the total N in seed increased by >50 kg N ha^−1^ (Supplementary Figure 7) compared with the 202 kg N^−1^ applied, indicating a low N use efficiency from this management practice. Further research is necessary for evaluating the efficiency and environmental impact of low N rates applied during seed growth in soybean.

### Effect of Inoculant Applications at R3

In this study, we evaluated the response of soybean yield and seed protein concentration to applications of liquid inoculant to the soil at beginning pod (R3) based on the results obtained by the study of Moretti et al. (2018). Our rationale was that elite strains of bacteria inoculated on seeds at sowing may have limited mobility to cause nodulation in lateral roots, which are major contributors to N_2_ fixation during seed fill (McDermott and Graham, 1989). The results showed that there is no significant effect of the additional inoculant application at R3 on soybean yield and seed composition, except soybean in 2019 following a cover crop at Kentucky. These results are in agreement with previous studies that found no effect of inoculant applications at planting (Ham et al., 1971; De Bruin et al., 2010; Carciochi et al., 2019) or from additional inoculant applications at R1 (Carciochi et al., 2019) in fields with the previous history of soybean cultivation. The study of Moretti et al. (2018) is one of the few studies evaluating supplemental inoculant spray applications after planting that found a significant effect on yield from this practice and in fields with previous soybean history. It is important to note that the soil pH in the study by Moretti et al. (2018) was 4.8 and that conventional seed inoculation at planting also increased yield relative to the control, despite high concentrations of *B. japonicum* in the soil. The study conducted by Moretti et al. (2018) found that additional inoculant applications from V1 to R1 increased yield compared with the seed inoculation at planting in one of the years of their study. Consistent with results from our study, the study of Moretti et al. (2018) did not find an effect of additional inoculant applications at R3 on soybean yield. Based on the results from our study and those by Moretti et al. (2018) it is unlikely that inoculant application at R3 or later could provide measurable benefits in fields with previous soybean history.

### Effect of Cover Crop

real cover crops can reduce N availability and cause yield reductions for rotational cereal grain crops (Singer and Kohler, 2005; Nielsen et al., 2016). Previous studies found no effect (Ruffo et al., 2004; Uchino et al., 2009; Pantoja et al., 2015; Wen et al., 2017; Acharya et al., 2020) or a negative effect (Eckert, 1988; Singer and Kohler, 2005; Harasim et al., 2017; Riedell et al., 2017) of cover crops on soybean yield. In contrast, the effect of cover crop rotations on soybean seed composition has not been extensively studied. We found that growing soybean after cereal cover crop with residue removed reduced yield in two out of six site years in this study. In addition, the effect of the cover crop showed an interaction with late-season input treatments and location, supporting our initial hypothesis. This was evident at the site in Minnesota in 2019, where the N fertilizer application increased yield in soybean after fallow, but not in soybean grown after a spring oat cover crop (Figure 3). The effect of the cover crop on soybean yield was further influenced by a cultivar effect, but that was not consistent across years or locations.

Limited information is available in the literature regarding the cover crop effect on soybean seed composition. We observed that cover crop decreased protein concentration on average (Figure 3B) and increased oil concentration in 2019 (Figure 4C) in our site in Minnesota. These results are in contrast with previous studies that found higher protein concentration (Harasim et al., 2017) and lower oil concentration (Singer and Kohler, 2005) in soybean grown after a cereal rye cover crop.

Overall, we found that cereal cover crops may have a negative effect on soybean yield and seed protein concentration in some cases, and reduce the yield response to late-season N fertilizer applications. In addition, these negative effects of the cover crop treatment were dependent on the location and more pronounced at the northernmost site in Minnesota. It is important to note that cover crop biomass was removed prior to soybean planting in our study to minimize N additions from cover crop residue mineralization. We were interested in testing the potential negative effects of low soil inorganic N after a cereal cover crop on soybean yield and seed composition. We found that N content in the aboveground cover crop biomass at termination (14–42 kg N ha^−1^) was always greater than the reduction in soil inorganic N in the top 30 cm of soil (up to 22 kg N ha^−1^ less than the control) (Table 3). Thus, managing cover crops in our trials by incorporating the residue or leaving it on the soil surface would return N to the soil, which could reduce some of the negative effects observed on yield and seed protein concentration. Moreover, the recovery and return of N after several years to the soil by non-harvested cover crops may lead to greater soil N supply and higher soybean yield and protein than a system without cover crops. However, these effects may not be evident in short-term cover crop rotations. We also observed a delay in the rate of development during late reproductive stages in Minnesota in soybean grown after a cover crop compared with fallow. This effect was not entirely attributed to a delay in emergence or vegetative stages due to possibly colder soil conditions after a cover crop relative to the fallow. Further research is necessary to study the long-term effects of cover crops on soybean yield components and seed composition.

### Potential to Increase Soybean Yield and Meal Protein With Cultural Practices

Although meal protein concentration is of high interest to the soybean industry, this is one of the few studies which documented the effect of agronomic practices on meal protein concentration. Meal protein concentration results from the combined effect of seed protein and oil concentration. Seed protein concentration is typically lower in northern U.S. states compared with southern latitudes (Hurburgh Jr et al., 1990; Rotundo et al., 2016; Naeve and Miller-Garvin, 2019). In addition, soybean produced in northern US latitudes has a tendency for relatively lower seed oil concentration compared with warmer locations in the south (Naeve and Miller-Garvin, 2019), contributing further to lowering meal protein concentration after oil extraction. Our results were consistent with this pattern. Soybean cultivars grown at our northernmost location in Minnesota did not meet the minimum concentration requirement of 440 mg g^−1^ of protein in soybean meal, with the exception of two cultivars in 2020 (Supplementary Figure 6). However, unlike seed oil concentration that showed a consistent effect of location, seed and meal protein concentration were more variable from year to year and across locations. The results from our study suggested that this variability may be partially associated with differences in soybean yield (Figure 6). For instance, the meal protein concentration in 2019 in Kentucky was below the minimum threshold in four out of six cultivars in 2019, and this location and year was coincident with the highest average yields in our study. Similarly, meal protein concentration was the highest on average at the site in Arkansas but still fell below the meal protein threshold on four occasions with full-season MG 4 cultivars (Supplementary Figure 6), which were the highest yielding cultivars at this location. Thus, our results suggested that low meal protein concentrations were partially determined by differences in environmental conditions and latitude that affected seed oil concentration, but also were largely affected by differences in yield that drive total seed N demand. This idea is supported by the negative asymptotic relationship that we found between meal protein concentration and yield (Figure 6). Late-season N fertilization was effective in increasing meal protein concentration on average and reducing the number of cases that did not meet the minimum meal protein concentration for high protein designation. However, growing soybean after a cover crop with residue removed reduced the efficacy of late-season N applications to some extent.

## Conclusions

There is limited information on the potential of cultural practices to increase seed protein concentration in soybean. We hypothesized that cultural practices that increase crop N availability during seed growth could have a positive effect on seed protein concentration. In particular, we evaluated the effect of liquid inoculant applications at R3, and N fertilizer applications after R5 on yield, seed and meal protein concentration. We found that inoculant applications did not affect soybean yield and seed composition in our fields with a previous history of soybean production. Furthermore, we found evidence that late-season N applications to soybean after the beginning of seed growth can be an effective tool to increase seed protein concentration in modern high-yielding cultivars. We also observed a yield increase in two out of three locations that provides scope for producers to address the declining trend in soybean seed protein concentration with economical rates of N fertilizer.

We hypothesized that the effect of late-season cultural practices on yield and seed protein concentration could depend on the location, on the crop yield potential as influenced by the cultivar maturity, and other factors that affect initial soil N availability such as cover crops. Our results partially supported this hypothesis. We found that growing soybean after a spring oat cover crop in Minnesota reduced the yield response to the N fertilizer treatment in one of the years at this location, compared with no cover crop. In addition, the positive effect of N fertilizer and the negative effect of cover crop on seed protein concentration were the most pronounced at the northernmost site in Minnesota. Cultivar maturity showed a main effect on yield and seed composition but did not influence the response to the N fertilizer application.

Low seed and meal protein concentrations were partially determined by an increase in latitude, but also variable from year to year and associated with high yields. Late-season N fertilizer was effective in reducing the number of cases that did not meet minimum meal protein requirements but relatively less effective in soybeans grown after a cover crop with biomass removed at termination. The potential benefits of long-term cover crop rotations without residue removed on soybean yield and seed composition remain to be investigated. Lastly, new studies need to determine the response of seed composition to low rates of N fertilizer as well as the environmental impact associated with this practice.

## Data Availability Statement

The original contributions presented in the study are included in the article/Supplementary Material, further inquiries can be directed to the corresponding author/s.

## Author Contributions

ERH, DH, SN, HP, LCP, and MS contributed to conception and design of the study. AC, SN, HP, LCP, and MS collected the data. AC and MS organized the database, performed the statistical analysis, and wrote the first draft of the manuscript. All authors contributed to critical manuscript revision, read, and approved the submitted version.

## Funding

The authors gratefully acknowledge the funding from United Soybean Board (USB Project# 1920-152-0127 and Project# 2020-152-0110) and from the University of Kentucky (Hatch funds) to conduct this research.

## Conflict of Interest

The authors declare that the research was conducted in the absence of any commercial or financial relationships that could be construed as a potential conflict of interest.

## Publisher's Note

All claims expressed in this article are solely those of the authors and do not necessarily represent those of their affiliated organizations, or those of the publisher, the editors and the reviewers. Any product that may be evaluated in this article, or claim that may be made by its manufacturer, is not guaranteed or endorsed by the publisher.
