# Supplementary material for: Late-Season Nitrogen Applications Increase Soybean Yield and Seed Protein Concentration
Source: Front Plant Sci. 2021 Oct 6;12:715940. doi: 10.3389/fpls.2021.715940 (PMC8527004; doi:10.3389/fpls.2021.715940)
Supplement: Supplementary file 1 [file Data_Sheet_1.docx]

**
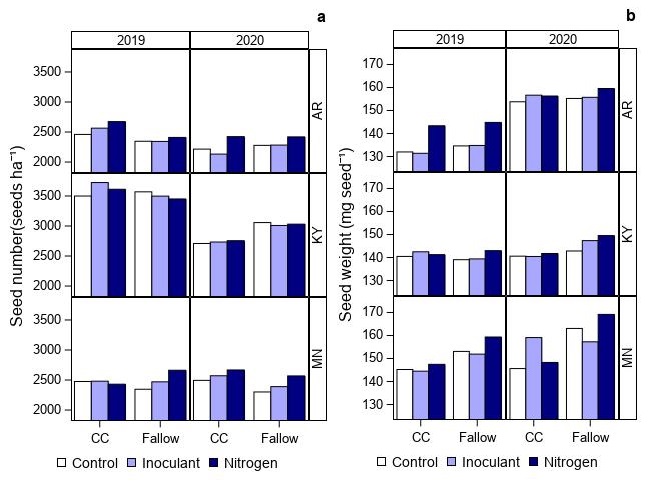
**

**Supplementary Figure 1.** Average seed number **(a)** and seed weight **(b)** by rotation type (CC, cover crop; or fallow) and late-season input treatment (Control, Inoculant application at R3, and N fertilizer applications after R5) at each year and location. Data averaged across cultivars.

**
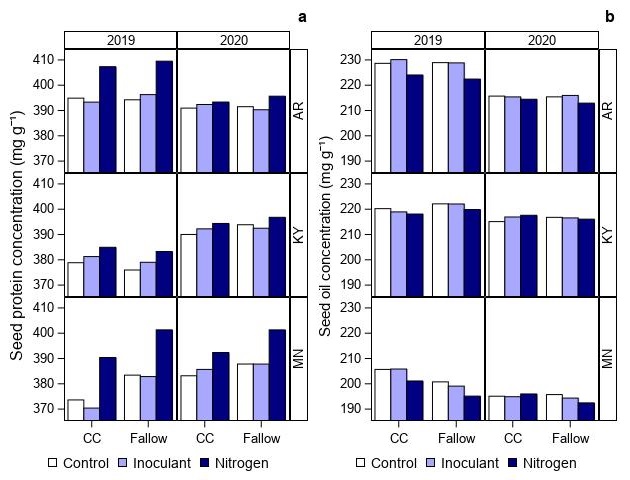
Supplementary Figure 2.** Average seed protein (a) and oil (b) concentration by rotation type (CC, cover crop; or fallow) and late-season input treatment (Control, Inoculant application at R3, and N fertilizer applications after R5) at each year and location. Data averaged across cultivars.

**
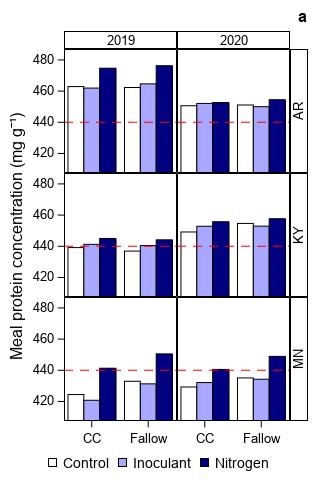
**

**Supplementary Figure 3.** Average meal protein concentration by rotation type (CC, cover crop; or fallow) and late-season input treatment (Control, Inoculant application at R3, and N fertilizer applications after R5) at each year and location. Data averaged across cultivars.

**
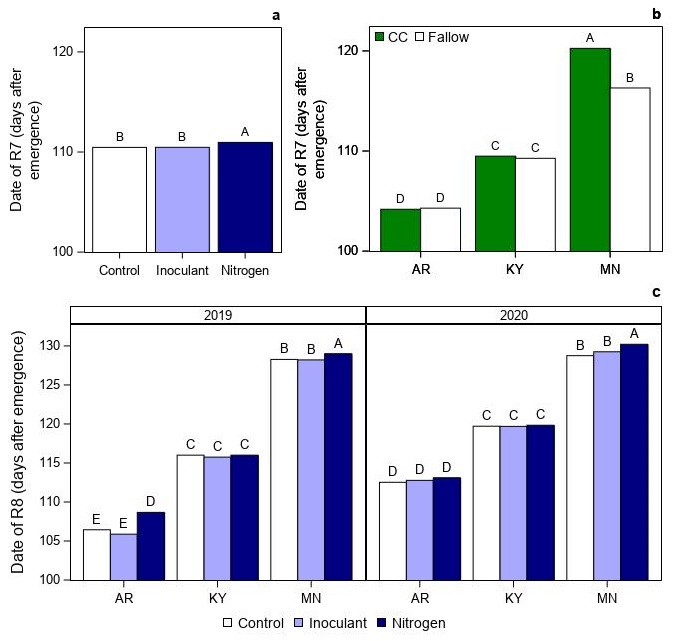
Supplementary Figure 4.** Days from emergence to physiological maturity (R7) by treatment **(a)** and by rotation type and location **(b).** Data averaged across years and cultivars. Days from emergence to harvest maturity (R8) by year, location, and treatment **(c).** Different letters on top of each bars indicate significantly different means at P<0.05.

**
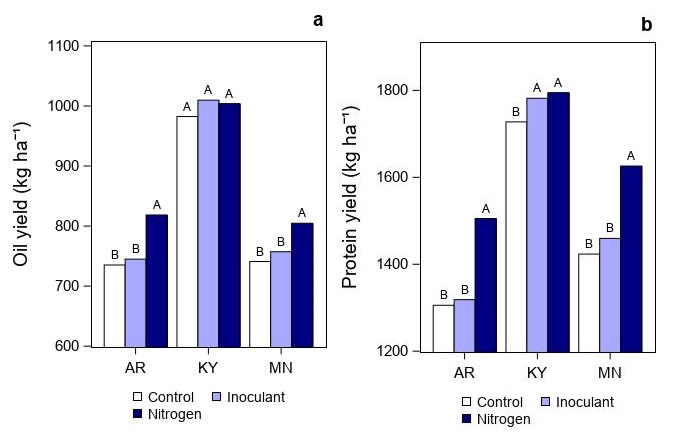
Supplementary Figure 5.** Mean total oil **(a)** and protein extractions in harvest seed **(b)** by late-season input treatment (Control, Inoculant application at R3, and N fertilizer applications after R5) at each location. Data averaged across years, rotation types, and cultivars. Different letters at the top of each bar indicate significantly different means at P<0.05.

**
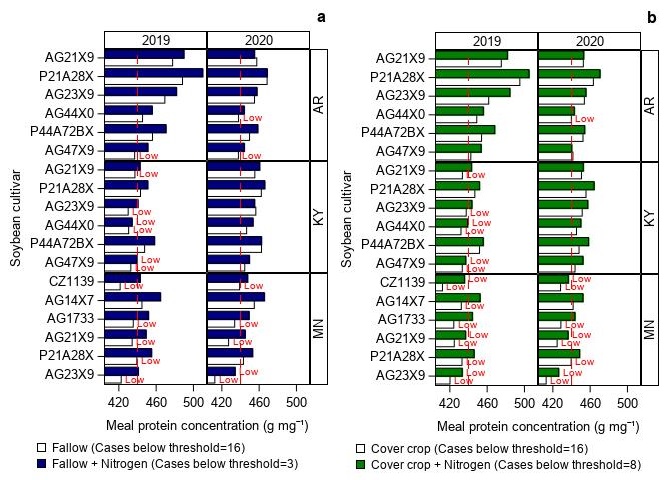
Supplementary Figure 6.** Meal protein concentration by cultivar in soybean grown after fallow (a) and soybean grown after a cover crop (b). The vertical red dashed line indicates the minimum threshold of 440 mg g^-1^ required by the meal industry for high protein meal designation. The letters “Low” indicate the cases when meal protein concentration fell below the threshold.

**
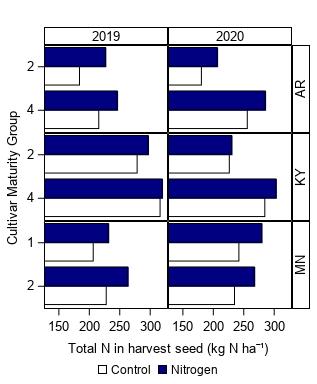
**

**Supplementary Figure 7.** Total N extractions in harvest seed (kg N ha^-1^) in control treatments and treatments receiving N fertilizer applications after R5. Data averaged across rotation type and cultivars.
